# *Antheraea* peptide and its analog: Their influence on the maturation of the reproductive system, embryogenesis, and early larval development in *Tenebrio molitor* L. beetle

**DOI:** 10.1371/journal.pone.0278473

**Published:** 2022-12-01

**Authors:** Karolina Walkowiak-Nowicka, Szymon Chowański, Joanna Pacholska-Bogalska, Zbigniew Adamski, Mariola Kuczer, Grzegorz Rosiński

**Affiliations:** 1 Department of Animal Physiology and Developmental Biology, Faculty of Biology, Adam Mickiewicz University, Poznań, Poland; 2 Laboratory of Electron and Confocal Microscopy, Faculty of Biology, Adam Mickiewicz University, Poznań, Poland; 3 Faculty of Chemistry, University of Wroclaw, Wrocław, Poland; USDA Agricultural Research Service, UNITED STATES

## Abstract

In recent years, many new immunologically active peptides from insects have been identified. Unfortunately, in most cases, their physiological functions are not fully known. One example is yamamarin, a pentapeptide isolated from the caterpillars of the *Antheraea yamamai* moth. This peptide has strong antiproliferative properties and is probably involved in the regulation of diapause. Additionally, antiviral activity was discovered. The results of the research presented in this paper are, to our knowledge, the first attempt to characterize the biological effects of yamamarin on the functioning of the reproductive processes and embryonic development of insects using a model species, the beetle *Tenebrio molitor*, a commonly known pest of grain storage. Simultaneously, we tested the possible activity of the molecule in an *in vivo* system. In this research, we present the multifaceted effects of yamamarin in this beetle. We show that yamamarin influences ovarian growth and development, maturation of terminal oocytes, level of *vitellogenin* gene transcript, the number of laid eggs, duration of embryonic development, and larval hatching. In experiments with palmitic acid-conjugated yamamarin (C16-yamamarin), we also showed that this peptide is a useful starting molecule for the synthesis of biopharmaceuticals or new peptidomimetics with gonadotropic activity and effects on embryonic development. The data obtained additionally provide new knowledge about the possible function of yamamarin in insect physiology, pointing to the important role of this pentapeptide as a regulator of reproductive processes and embryonic development in a heterologous bioassay with *T*. *molitor*.

## Introduction

Yamamarin is a peptide (H-Asp-Ile-Leu-Arg-Gly-NH_2_) isolated from the hemolymph of *Antheraea yamamai* caterpillars [1]. However, what is its physiological function? Research, that has focused on defining the physiological role of those peptides in insects has thus shown an effect on the immune system (*e*.*g*., increasing the activity of prophenoloxidase, causing changes in the hemocytes *F*-actin cytoskeleton and stimulating phagocytic activity) [2], myocardial activity (cardioinhibitory effect) in the beetle *Tenebrio molitor* [3] and inhibition of the process of cellular respiration and the cell cycle in *Drosophila Schneider S2* cells and caused growth arrest in murine leukemic cells [4]. Yamamarin also appears to be involved in the regulation of diapause and/or development; for example, it induces the initiation of diapause in the moth *Bombyx mori* [5]. Yang, Abe [5] injected yamamarin and palmitic acid-conjugated yamamarin (C16-yamamarin) into the pupae of females who were previously experimentally programmed to lay non diapause eggs. After eclosion females oviposited eggs with a high incidence of diapause. Additionally, yamamarin inhibits embryonic development in *B*. *mori* [4, 5]. Interestingly, the palmitoyl derivative of yamamarin showed 20-fold higher activity than pure yamamarin [5]. Yamamarin also has an inhibitory effect *in vitro* on the growth of *A*. *yamamai* ovary cell line cells, *Drosophila melanogaster* S2 cell line cells and rat tumor cells, which confirms its anti-proliferative activity [5]. In the presence of yamamarin, the growth of cells was slower, and a 23.6% reduction in proliferation was detected [4]. All tricarboxylic acid cycle-related genes were reported to be downregulated, resulting in a reduction in the mitochondrial respiration rate [4, 6]. Some studies have also shown that yamamarin may have antiviral effects, inhibiting the replication of the human herpesvirus 1 McIntyre strain (HHV-1MC) virus *in vitro* at maximum nontoxic concentrations. Similar effects were observed for yamamarin modified at the *N*-terminal in which the Asp residue was substituted by Arg, Gly, Ala, Asn or Gln [5, 7]. Although the wide range of effects that yamamarin causes becomes increasingly apparent, its physiological role and mode of action are fragmented and not fully understood. Considering the potential use of yamamarin and C16-yamamarin, it seems reasonable to select for tests the reproductive organs, as a place of intense cell divisions. The possible role of both peptides as a regulators of reproduction and development makes them good candidates for further studies concerning chemical modifications of peptides and their possible role as regulators might also serve as a model for the synthesis of alternatives to synthetic insecticides, replacing traditional synthetic insecticides with low species specificity and highly harmful effects on nontarget organisms (so-called peptidomimetics) or may shed light on the synthesis of a new potential pharmaceutical *e*.*g*., which reduces metabolism or has anticancer activity, as its activity seems to be correlated with inhibition/reduction of cell proliferation. Nevertheless, because native peptide compounds have quite low stability in the natural environment [8], to increase their stability and enhance their ability to penetrate the cell membrane, we decided to modify the yamamarin structure by coupling it with a long-chain fatty acid, palmitic acid.

However, considering the data about the strong antiproliferative properties of yamamarin and its effects on diapausing pupae of *B*. *mori*, we were interested in how this peptide affects insect reproduction processes from vitellogenesis through oocyte maturation, oviposition, and embryonic development up to larval hatching. The female reproductive system of the *T*. *molitor* beetle consists of meroistic-telotrophic ovaries, which are functionally classified as asynchronous ovaries, which means oocytes do not grow simultaneously in all ovarioles [9]. Ovarian growth finishes in approximately 2 days, and maturation of oocytes occurs in the first few days (approximately 6 days) after the insects become imagos [10]. Therefore, to assess the effect of peptides on the formation and development of ovaries and to compare the results obtained, we focused on the assessment of parameters in females during the first ovarian cycle, *i*.*e*., during the first 8 days after the transition to the imago stage [11].

## Materials and methods

### Insects

A stock culture of *T*. *molitor* L. was maintained at the Department of Animal Physiology and Developmental Biology, Adam Mickiewicz University in Poznań, as described previously by Rosiński, Wrzeszcz [12]. The culture was kept at constant temperature (+28°C), relative humidity (65%), and photoperiod 8 h light to 16 h dark, with constant access to food, which is flour (10 g per container) and a water source—carrot slices (5 slices per container). The control and experimental groups were kept under the same temperature, relative humidity, and photoperiod conditions. The sex of insects was distinguished in the pupal stage. Males were tagged and placed in the container together with females in a 1:1 ratio. No approval of research ethics committees was required to accomplish the goals of this study because experimental work was conducted with an unregulated invertebrate species.

### Tested compounds

For bioassays, stock solutions of yamamarin and its analog (yamamarin conjugated with palmitic acid (C16-yamamarin)) were prepared in sterile conditions by dissolving in physiological saline for *T*. *molitor* (274 mM NaCl, 19 mM KCl, 9 mM CaCl_2_, pH 7.0) and were stored at -80°C. In the case of experiments with topical application on eggs, the peptides were dissolved in double-distilled water immediately prior to their topical application to the eggs to avoid the crystallization of salts on the surface of eggs.

### Measurement of hemolymph volume

Based on the work of Tabunoki, Dittmer [13], we prepared a test tube sieve. A small hole was made in the bottom of the Eppendorf test tube with a capacity of 0.5 mL, and the tube was placed in a larger test tube (1.5 mL). After anesthesia with endogenous CO_2_ (by immersing the test tube in a beaker with water for 7 minutes), a fragment of the antennae (flagellum) and all legs (tibia level) of adult imago beetle were cut off. Then, insects were placed in the previously prepared test tube “sieve” and immediately centrifuged at 22°C for 25 min, at 3,000 x *g* to avoid coagulation of hemolymph. Then, the volume of the obtained hemolymph was measured. The study showed that *T*. *molitor* insects have approximately 18 μL (with a mean value of 17.78 μL and a median value of 17.75 μL; SD = 3.086) of hemolymph. Forty individuals were used in the experiment.

### Injections

**Fig 1:**
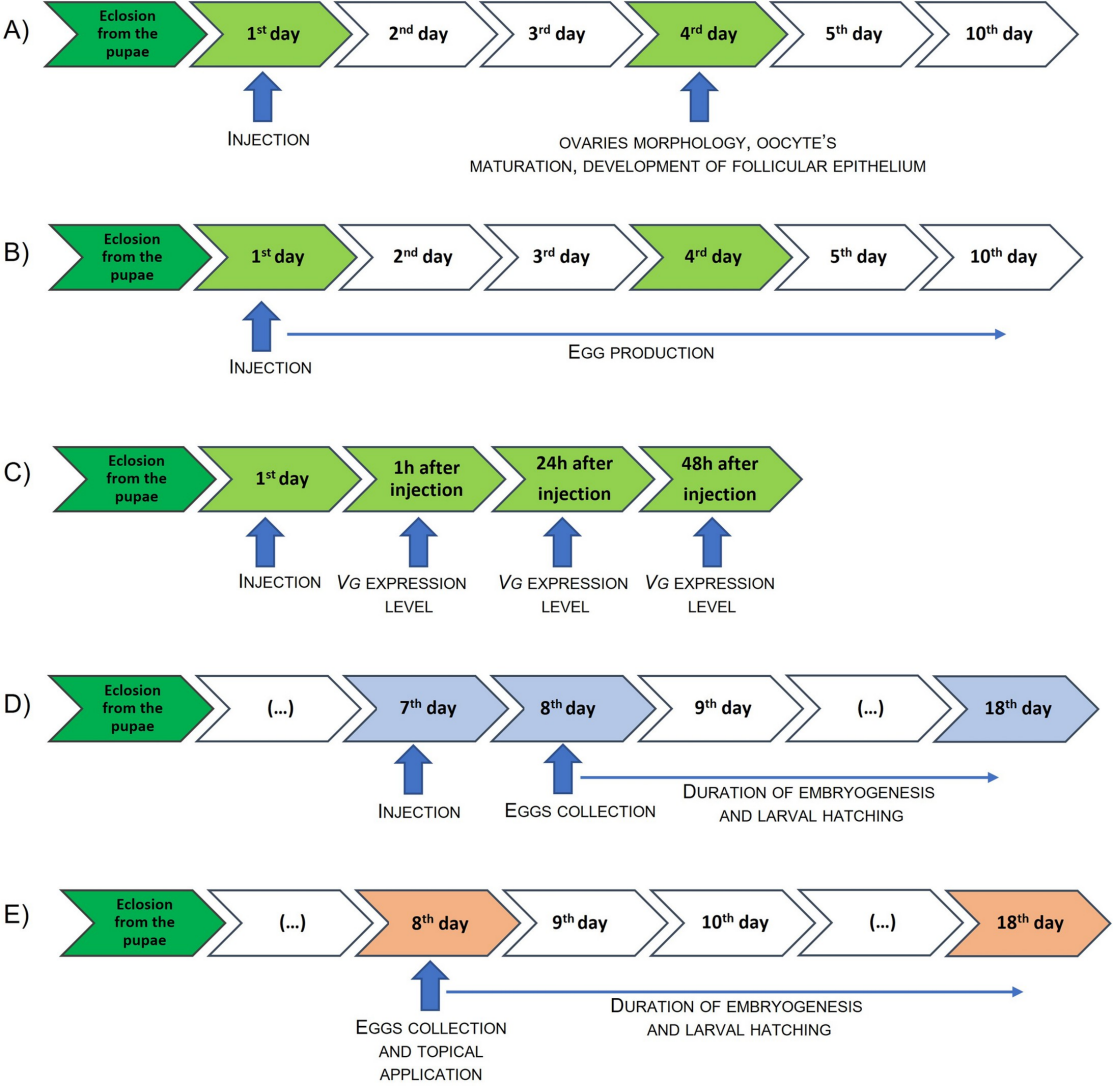
Timeline of experiments. A—Gonadotropic effects of peptides in females during the first reproduction cycle, the influence of peptides on the oocyte’s maturation process and development of follicular epithelium development, B—Influence of peptides on egg production, C—Examination of gene encoding vitellogenin expression level, D—Duration of embryogenesis and larval hatching after peptideinjection, E—Duration of embryogenesis and larval hatching after topical application of peptides.

Control insects were injected with physiological saline, and in experiments with topical application, the control eggs were treated with double-distilled water. On the first day after eclosion (Fig 1), the females were injected with peptide solutions with concentrations of 10^−11^, 10^−7^, and 10^−3^ M in a volume of 2 μL to obtain their final concentrations in the hemolymph approximately equal to 10^−13^, 10^−9^, and 10^−5^ M respectively. The tested compounds were applied by injection through the ventral membrane, between the thorax and abdomen, using a Hamilton syringe (Hamilton Company). Before injection, insects were anesthetized with endogenic CO_2_ by immersion in a beaker of water for 7 minutes, disinfected with 70% ethanol (by short-lived immersion in a beaker with ethanol), washed with Milli-Q water, and dried on tissue-paper.

For comparison, in the test using the topical application of compounds, we used, the ‘final’ concentrations, *i*.*e*., 10^−13^, 10^−9^ and 10^−5^ M, so that the obtained results could be compared with the results obtained after injection.

In our research to assess the effect of peptides on the development of ovaries and reproduction (considering the asynchrony of the ovary), the injection of females took place on the day following the transition to the imago stage (eclosion). For topical application of peptides and its effect, to reduce the false-negative effect, we conducted tests with topical application only on fertilized eggs. As *T*. *molitor* begins the copulatory events on the 2^nd^ day after the imago transition and the egg develops approximately 6 days, on the ‘collection date’ we chose 8 days after the imago transition [14]. Therefore, eggs were collected from females on the 8^th^ day after the transition to the imago stage.

### Yamamarin and palmitic acid-conjugated yamamarin synthesis

Yamamarin and palmitic acid-conjugated yamamarin (C16-yamamarin) synthesis was performed manually with a solid phase method according to a standard 9-fluorenylmethoxycarbonyl (Fmoc) procedure [15]. Amino acids were assembled on the MBHA-Rink amide resin. As a coupling reagent, 2(1Hbenzotriazole-1-yl)-1,1,3,3-tetramethyluronium hexafluorophosphate (HBTU) in the presence of 1-hydroxybenzotriazole (HOBt) was used. The N-Fmoc group was removed with 20% piperidine in *N*,*N*-dimethylformamide (DMF). The peptide-resin was cleaved from the resin using a mixture of trifluoroacetic acid/water/triisopropylsilane (95:2.5:2.5) at room temperature for 2 h. The crude compound was dissolved in water, lyophilized, and purified by reversed phase–high performance liquid chromatography (RP-HPLC). Yamamarin and its analog were purified by preparative RP-HPLC on a Varian ProStar column–Tosoh Biosciences ODS-120T C18 (ODS 300 x 21.5 mm). Water-acetonitrile gradients containing 0.1% trifluoroacetic acid (TFA) at a flow rate of 7 mL/min were used for purification with UV detection at 210/254 nm. Analytical HPLC was performed using Thermo Separation Products with a Vydac ProteinRP C18 column (4.6 mm × 250 mm) (Grace, Deerfield, IL, USA) and a linear gradient from 0 to 100% B in 60 min, a flow rate of 1 mL/min, solvent A = 0.1% TFA in water, solvent B = 0.1% TFA in 80% acetonitrile/water, and UV detection at 210 nm. The molecular weights of the synthesized yamamarin and 16-yamamarin were confirmed by electrospray ionization–mass spectrometry (ESI-MS) using an Apex-Qe Ultra 7T Fourier transform–ion cyclotron resonance (FT-ICR) instrument (Bruker Daltonic, Bremen, Germany).

### Ovaries isolation technique

After insects were anesthetized with endogenic CO_2_ by immersion for 7 min in a beaker with water, the ovaries were isolated with microsurgical instruments under a stereoscopic microscope. First, insects were decapitated, and then the wings, legs and integument of the abdomen were removed. Then, ovaries were dissected with microsurgical tweezers, cleaned of fragments of the fat body tissue and Malpighian tubules, and finally placed in physiological saline.

### Gonadotropic effects of peptides in females during the first reproduction cycle

Females were injected with the tested peptide on the first day after eclosion. On the 3^rd^ day after injection, the ovaries with oviducts were isolated (see ovary isolation technique) and then fixed in 4% paraformaldehyde for 10 min. Evaluation of the general morphology of ovaries was performed using an Olympus SZX12 stereomicroscope.

### Influence of peptides on the oocyte maturation process

When an oocyte moves into the transitional zone it becomes surrounded by prefollicular cells which ultimately change into follicular epithelium cells. Follicular epithelial cells change their shape into more cubical and spaces between them occurs (the intercellular spaces phase, the so-called *patency* phase), and the intensive, rapid growth of oocytes starts. When the oocyte reaches the final size, the connection with the germarium completes its action. Follicular epithelial cells flatten, the spaces between them disappear, yolk deposition stops, and cells start to secrete chorion [9]. The effect of peptides on the growth and maturation process of the oocytes was assessed by measuring the size of terminal oocytes using the Olympus SZX12 stereomicroscope. Measurements of the oocyte length and width were performed using an image analysis Olympus DP-Soft (Analysis, version 3.0). The volume of the terminal oocytes was calculated from the formula for the rotational extended ellipsoid:

$$\text{V}=\frac{4}{3}{\pi \text{ab}}^{2}$$

where *a*–length of terminal oocyte, *b*–width of terminal oocyte.

### Influence of peptides on the development of follicular epithelium

When yolk synthesis and deposition are about to start, follicular epithelial cells change their shape to become more cubical, and spaces between follicular cells appear. This step is mandatory for yolk passage from the place where it was synthesized, so from fat body tissue, into the oocyte. The development of follicular epithelium was examined on the fourth day of the first reproductive cycle (3 days after injection of the peptides, the time was previously assessed). At that point, the *patency* is best visible. The *patency* was experimentally examined, and this stage falls on the 4^th^ day after emergence in the imago stage. To visualize the intercellular spaces in the follicular epithelium, isolated ovaries were fixed with 4% paraformaldehyde and stained with 1% Evans blue according to the procedure described by Czarniewska, Mrówczynska [16]. The assessment of follicular epithelium development was performed using a Stereo Lumar.V12 stereoscopic microscope (Zeiss).

**Fig 2:**
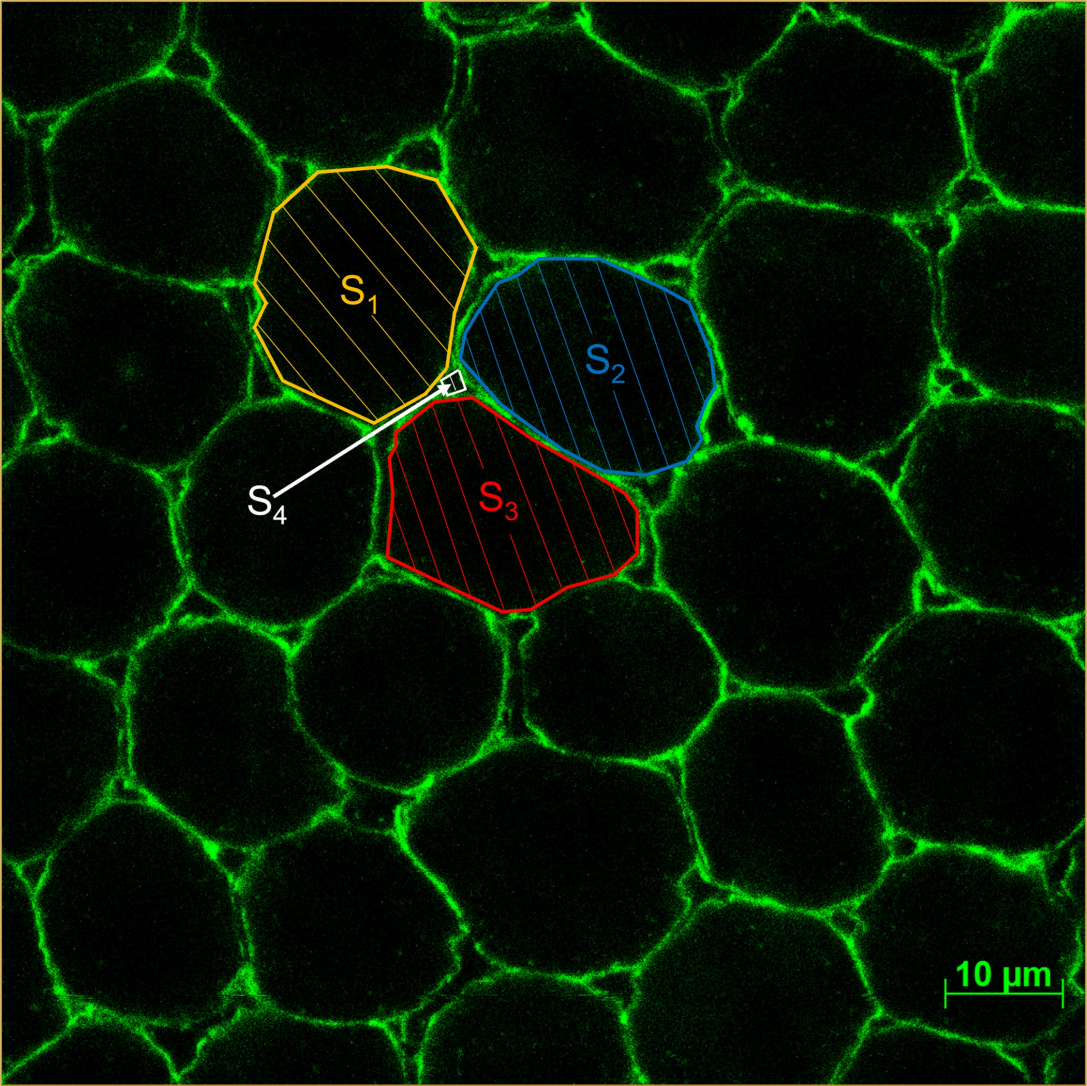
Scheme of follicular epithelium and method of its analysis. S_1_–S_3_ follicular cells, S_4_–intercellular space.

The effects of yamamarin and C16-yamamarin on the development of the follicular epithelium were also checked using fluorescent dyes under a confocal microscope (Fig 2). In each variant, five ovaries (from five females), on which three random pictures were taken, were analyzed. Visualization of the *F*-actin cytoskeleton which allows to visualization of the intercellular spaces in the follicular epithelium was performed on isolated ovaries in accordance with Czarniewska et al. [17]. Isolated ovaries were fixed with a 4% solution of paraformaldehyde in physiological saline and then permeabilized with 0.1% Triton-X 100 (Sigma-Aldrich). Staining of follicular epithelium cells *F*-actin cytoskeleton was made with Oregon Green® 488 phalloidin (Invitrogen) in 1% of bovine serum albumin (BSA; prior dissolved in PS_A_) for 20 min in the dark at room temperature. Next, stained ovaries were rinsed in PS_A_, placed on microscopic slides in mixture of glycerol, phosphate buffered saline (PBS) and triethylenediamine (DABCO) and covered with cover slides. The assessment of follicular epithelium development was performed using Carl Zeiss LSM 510 confocal microscopes with AxioVision SE64 Rel. 4.9.1 program.

The *patency* index and the size of intercellular spaces were measured according to the procedure described by Pszczółkowski [18] adjusted to follicular epithelium of *T*. *molitor*. In experiments, we measured: 1) the size of intercellular spaces, by averaging the measurement of the surface area of all intercellular spaces visible on microscope image (S_4_) in each of the tested variants 2) the surface of follicular cells (S_1_, S_2_, S_3_) by averaging the measurement of the surface area of all follicular cells visible on microscope image in each of the tested variants, and 3) *patency* based on the surface area of the follicular cells (S_1_, S_2_, S_3_) tangent to a specific intercellular space (Fig 2) and the surface area of the intercellular space (S_4_) and calculated with the formula:

$$P=\frac{S_{4}}{\left(S_{1}+S_{2}+S_{3}\right)}$$

### Examination of the gene encoding vitellogenin expression

Fat body tissue was isolated from females injected on the first day after eclosion with a 10^−7^ M solution of yamamarin (used in this experiment because of the greatest effects that peptide at this concentration caused on the size of terminal oocytes) and physiological saline was used as a control. Tissue isolation was performed 1 h, 24 h, and 48 h after injection. Tissues were immediately placed in lysing reagent (Zymo Research) and homogenized with a pestle. Before RNA isolation, samples were stored at -80°C. Total RNA was isolated using the ready-made Quick-RNA™ MiniPrep kit (Zymo Research) according to the manufacturer’s instructions. The samples were then treated with DNase I (Thermo Scientific) to remove residual DNA. RNA concentration and purity were determined spectrophotometrically using a Synergy H1 Hybrid Plate Reader (Biotek). The obtained RNA was used for cDNA synthesis. RNA (500 ng) was used as a template to perform the reverse transcription reaction using the ready-made RevertAid™ Reverse Transcriptase kit (Thermo Scientific) as described by the manufacturer. The obtained cDNA samples were stored at -80°C and used for qPCR. The analysis included “no template control” and “no RT control” reactions to ensure that there was no contamination with foreign DNA or genomic DNA contamination. The primers (Table 1) were designed based on sequences available in public databases (National Center for Biotechnology Information, NCBI) (accession number: AY714212.2) using Primer3 software, which is part of the Geneious 9.1.8 package. The synthesis of primers was carried out at the Institute of Biochemistry and Biophysics of the Polish Academy of Sciences in Warsaw.

**Table 1:** Sequences of primers used in qPCR. The sequences of the primers based on data published by Lord and coworkers [19] are marked with *.

Name	Sequence	Amplicon length
Vito-F	5’ GTTTATAAAGTTCATAGTAATGCAGA 3’	181 bp
Vito-R	5’ GGATATAAACGTGAATTTTTCTAGTA 3’	
*Syn1-F	5’ GGCTTCATGGATGCATTTTT 3’	200 bp
*Syn1-R	5’ TTAAGCTTGGCACGGACTTT 3’	

The cDNA served as a matrix in a PCR for amplification of the *vitellogenin* gene fragment using specific primers. The PCR conditions for the amplified gene were determined and optimized. The final mixture contained 1 μM primers, 200 μM dNTP, 1x PCR buffer and 1 U/25 μL DreamTaq Polymerase mixture (Thermo Scientific). Samples were amplified in 34 cycles: denaturation at 95°C for 30 seconds, hybridization at 51°C for 30 seconds, and product extension at 72°C for 1 min in a DNA thermocycler (Biometra). The amplification products were then separated on a 2% agarose gel with the addition of ethidium bromide and visualized in a UV transilluminator. The PCR products were then isolated from the agarose gel using the Zymoclean™ Gel DNA Recovery Kit (Zymo Research) according to the manufacturer’s instructions. The DNA fragments purified from the agarose gel were then sequenced in the Sequencing and Molecular Biology Laboratory of the Faculty of Biology at the University of Adam Mickiewicz University in Poznań. To confirm that the obtained PCR product is a coding sequence for the *vitellogenin* gene, the obtained results were compared with sequences deposited in public databases using the Chromas Lite 2.01 and basic alignment search tool (BLAST) programs (http://blast.ncbi.nlm.nih.gov/Blast.cgi).

qPCR was conducted in a RotorGene 6000 (Corbett Research) using SYBR-Green I as the detection dye. The amplification was performed with specific primers with stable expression in fat body tissue listed in Table 1 and the quantity of the vitellogenin transcripts in each sample was standardized by *syntaxin-1* transcript level. qPCRs were performed separately for the investigated *vitellogenin* gene and for the reference gene in a total volume of 20 μL. Two microliters of cDNA was added to an 18 μL mixture of Power SYBR Green Master Mix (Applied Biosystems) and primers. Forty cycles of reaction were performed under the following conditions: hold at 50°C for 2 min, denaturation at 95°C for 2 min, annealing and elongation at 60°C for 60 s. Standard curves with five successive tenfold dilutions of the linear DNA product of the given primer set were constructed to determine the levels of transcript data in the test samples. Data were collected using RotorGene 6000 software (Corbett Research). Target cDNA was quantified using the ΔΔ*Ct* method [20]. The results are presented in a bar graph in which error bars refers to the original *Ct* values. Each biological sample (4 independent samples for each variant) consisted of tissue isolated from ≥ 10 females. Three technical repetitions were made.

### Influence of peptides on egg production, embryogenesis duration and larval hatching

One-day-old females were injected with yamamarin or its conjugate on the first day of the first reproductive cycle. Then females and males were placed in plastic containers (diameter 10 cm, with a density of 10 individuals– 5 females + 5 males) and were fed with coarse grain flour, with the addition of 5 slices of fresh carrot. Flour was also a breeding medium, to imitate the conditions in which they live, and additionally, eggs attached to the culture medium by a sticky secretion of the collateral glands, were easy to collect. Each day, for ten days the number of eggs laid by females in the culture medium and stuck into the plastic box surfaces was counted in the test and control groups. The eggs were collected from the females at the same time each day. The eggs were isolated from the culture medium using the procedure previously described in the patent study (PL Nr 217868B1) by Rosiński [21] and briefly passed through a sieve with a hole diameter of 1.15 mm. The culture medium was changed each day after egg collection. In a study of the effect of yamamarin and its analog on the embryonic development of a new generation, eggs collected from 8-day-old females injected on the 7^th^ day with these peptides and mated with males on the first day after eclosion, were used. The condition of the eggs was checked under a stereoscopic microscope. Well-developed, undamaged (no dents, even in color and of equal size) eggs were chosen. The eggs were placed in Petri dishes with a small amount of coarse grain flour and kept in an incubator (Memmert INE 400) at a constant temperature of 28°C and 65% relative humidity. For the next 10 days, the condition of eggs and the number of hatching larvae were counted. The time was chosen based on previous observations during which all control larvae hatch within 10 days.

### Impact of topical application of peptides on embryonal development and larval hatching

One-day-old females were placed with males (5 females + 5 males) in a plastic box (diameter: 10 cm). Eggs laid by 8-day-old females were separated from the breeding medium and were prepared according to the method described in the patent procedure [21]. Then, eggs were cleaned from left-over flour with a water stream (25°C). In the next step, the egg condition was checked under a stereoscopic microscope. Well-developed, undamaged eggs were chosen, manually placed on a transparent dish with a smooth surface, and then flooded for 15 seconds with 20 microliters of peptide of known concentration (*per* egg). After this time, excess fluid was gently aspirated with a pipette, and then the eggs were dried by calmly touching the substrate with tissue paper. To avoid crystallization of salt on the surface of the egg (chorion or aeropyle) and degeneration of eggs, water solutions or water as control were used instead of saline. Then, the eggs were placed in an incubator with constant temperature (28°C) and humidity (60% ± 5%), and the number of hatched larvae was counted within 10 days. The time was chosen based on previous observations during which all control larvae hatch within 10 days.

### Statistical analysis

For the statistical analysis of the obtained data, we used GraphPad Prism (version 8) software (Department of Animal Physiology and Developmental Biology AMU license). Before performing the analysis, we used the Shapiro-Wilk test to assay the normality of the distribution. To check the homogeneity of variance, Levene’s test was used. For statistical comparison of the groups with normal distribution, one-way analysis of variance (ANOVA) with Dunn’s multiple comparison test and two-way ANOVA with Dunnett’s multiple comparison test were used, and for nonparametric data, the Kruskal-Wallis test was used. In addition, Student’s *t*-test and, for nonparametric data, Mann-Whitney U test were used. Spearman’s correlation coefficient was also used. The data shown are the mean value of the parameter ± SD.

## Results

### The gonadotropic effect of the peptides during the first reproductive cycle

**Fig 3:**
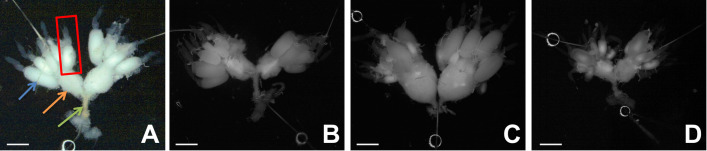
Representative micrographs obtained with a stereoscopic microscope of 4-day-old *T*. *molitor* ovaries isolated from control females injected with physiological saline (B), yamamarin (C) and C16-yamamarin (D) at concentrations of 10^−7^ M. (A) Representative picture of ovary: red frame—single ovariole, blue arrow—terminal oocyte, orange arrow—lateral oviduct, green arrow—common oviduct. The white scale bar corresponds to 1000 μm.

Injection of yamamarin into female *T*. *molitor* during the first reproductive cycle caused changes in the development and morphology of the ovaries. The gonadotropic effect of yamamarin in females of this beetle is multifaceted. Yamamarin had a stimulating effect on ovarian development with the simultaneous acceleration of the growth and maturation of terminal oocytes (Fig 3).

**Fig 4:**
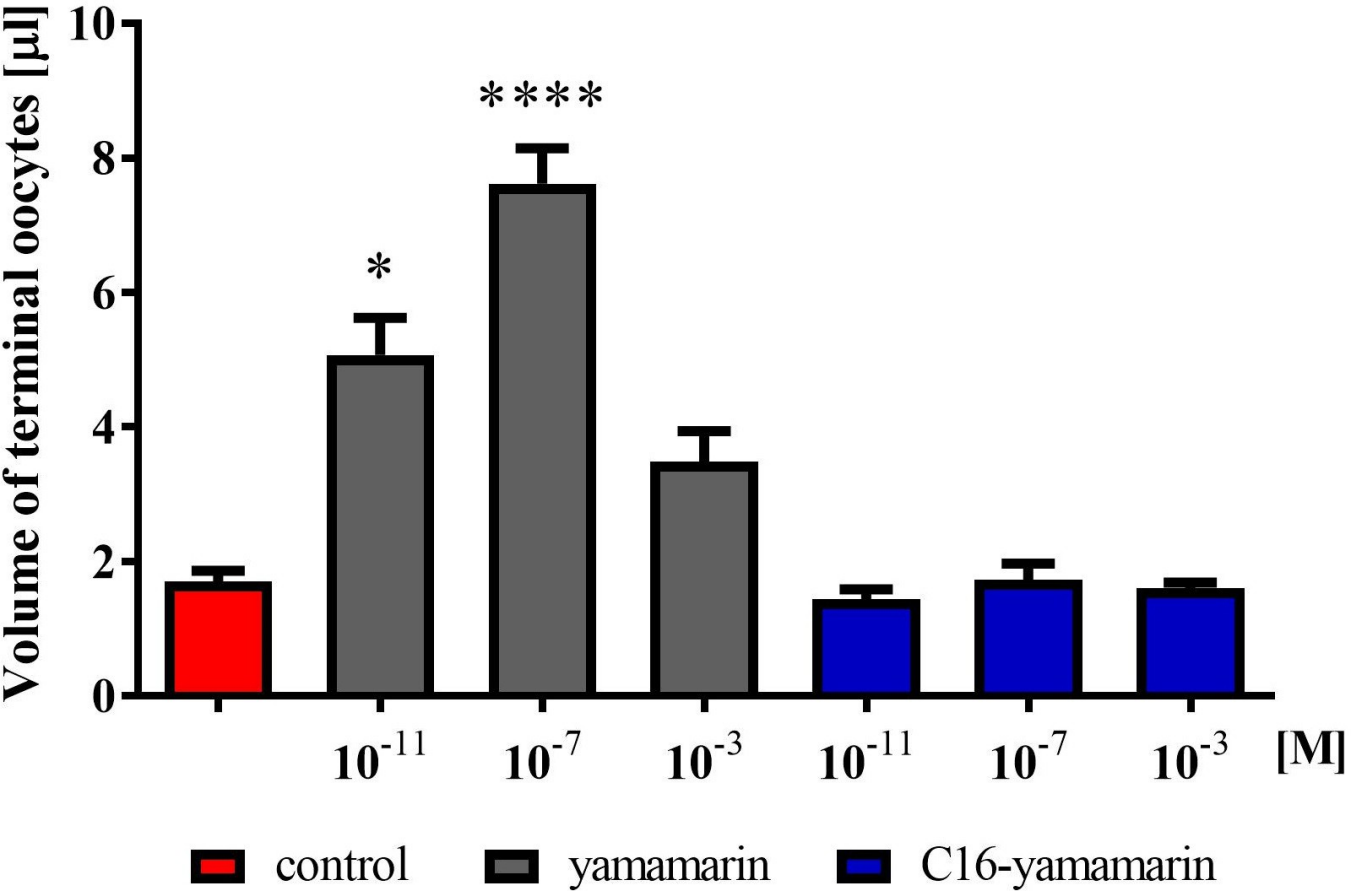
Changes in the volume of the terminal oocytes in the 4-day-old *T*. *molitor* females, injected on the first day after eclosion with saline (control), yamamarin, and C16-yamamarin at concentrations 10^−11^, 10^−7^ and 10^−3^ M. Data represent the mean value ± SD for *n* ≥ 10. Statistically significant differences (one-way ANOVA, Dunn’s multiple comparisons test) from the control values are indicated by asterisks as indicated: *p* ≤ 0.0001 (****), *p* ≤ 0.1 (*).

After the injection of the peptide at the tested concentrations (10^−11^, 10^−7^, 10^−3^ M), the increased ovulation process of mature eggs into the lateral oviducts was observed, which resulted in their significant enlargement. Contrary to the action of yamamarin, all three injected concentrations of C16-yamamarin did not induce accelerated development of terminal oocytes or their increased ovulation into the lateral oviduct. After injection of this analog into 4-day-old females, no mature eggs were found in the lateral oviducts (Fig 3). We also analyzed the volume of terminal oocytes (Kruskal-Wallis, H = 49.07; *p* ≤ 0.0001) (Fig 4). In the control females, the average volume of terminal oocytes was 1.7 μL, whereas in females injected with yamamarin at the tested concentrations, a significant increase in the terminal oocyte volume was statistically significant only at the lower peptide concentration, and when the peptide was applied at a concentration of 10^−3^ M the changes were not statistically significant (Fig 4). The average volume of terminal oocytes after the application of yamamarin at concentrations of 10^−11^ (mean 5.0 μL), 10^−7^ M (mean 7.6 μL) and 10^−3^ M (mean 3.5 μL) was 2.9, 4.5 and 2.0 times higher, respectively, than that in the control females. Interestingly, injections of the C16-yamamarin analog, did not change the volume of terminal oocytes. The volume of terminal oocytes after injection of each of the three tested concentrations (10^−11^, 10^−7^, 10^−3^ M mean value 1.4 μL, 1.7 μL and 1.6 μL respectively) of this analog was similar to the volume of terminal oocytes of control females.

### Impact of peptides on follicular epithelium development

**Fig 5:**
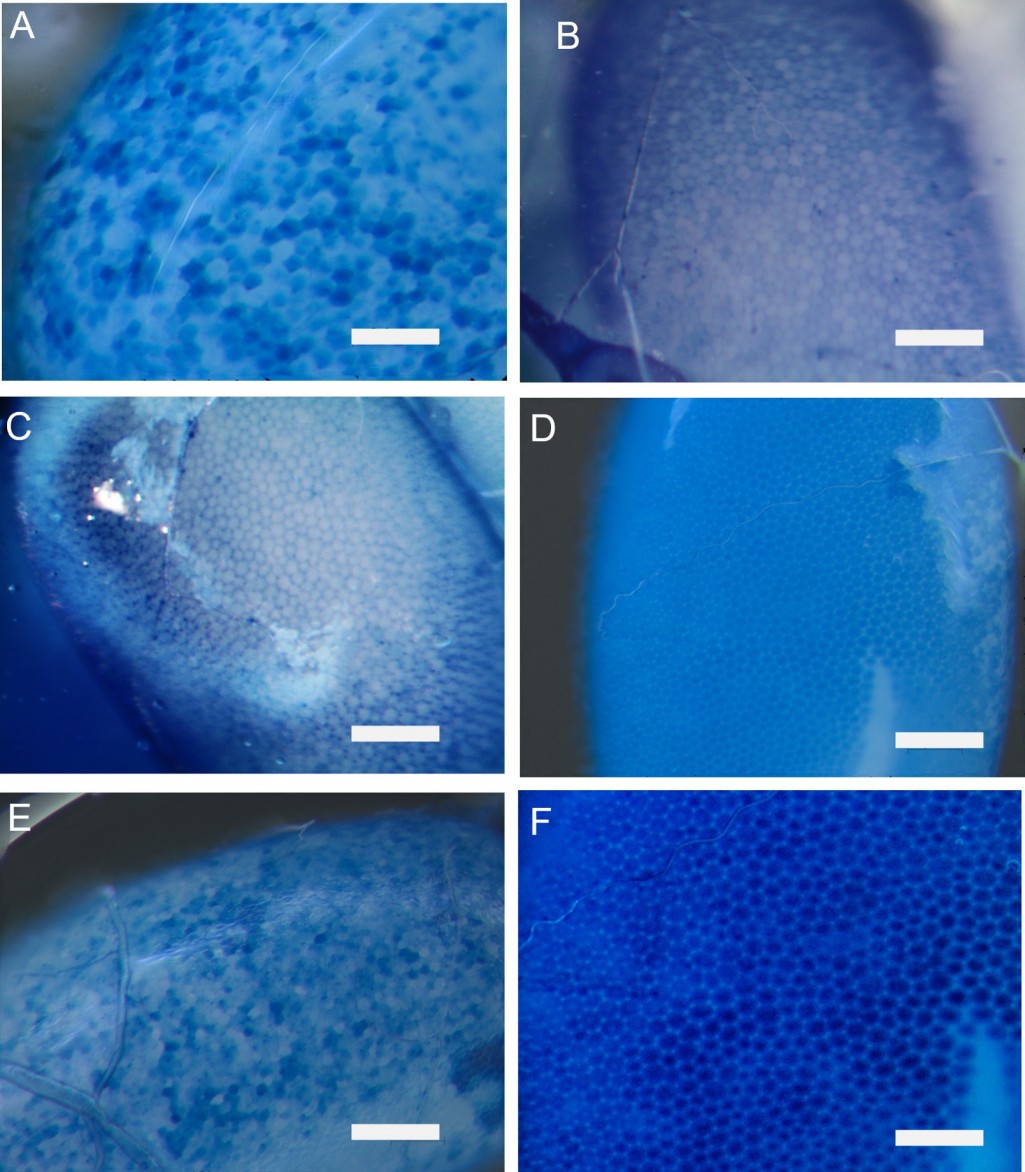
Representative images of a properly developed follicular epithelium in ovaries isolated from 4-day-old *T*. *molitor* beetles injected on the first day after eclosion with saline (A, B), yamamarin (C, D) and C16-yamamarin (E, F) at concentrations of 10^−3^ M. The white scale bar corresponds to 100 μm.

A well-developed follicular epithelium with clear intercellular spaces between epithelial cells (*patency*) was observed in ovaries isolated from 4-day-old control females injected with physiological solution. In both, yamamarin and C16-yamamarin, we observed properly formed epithelial cells and well-developed intracellular spaces (*patency*) (Fig 5).

**Fig 6:**
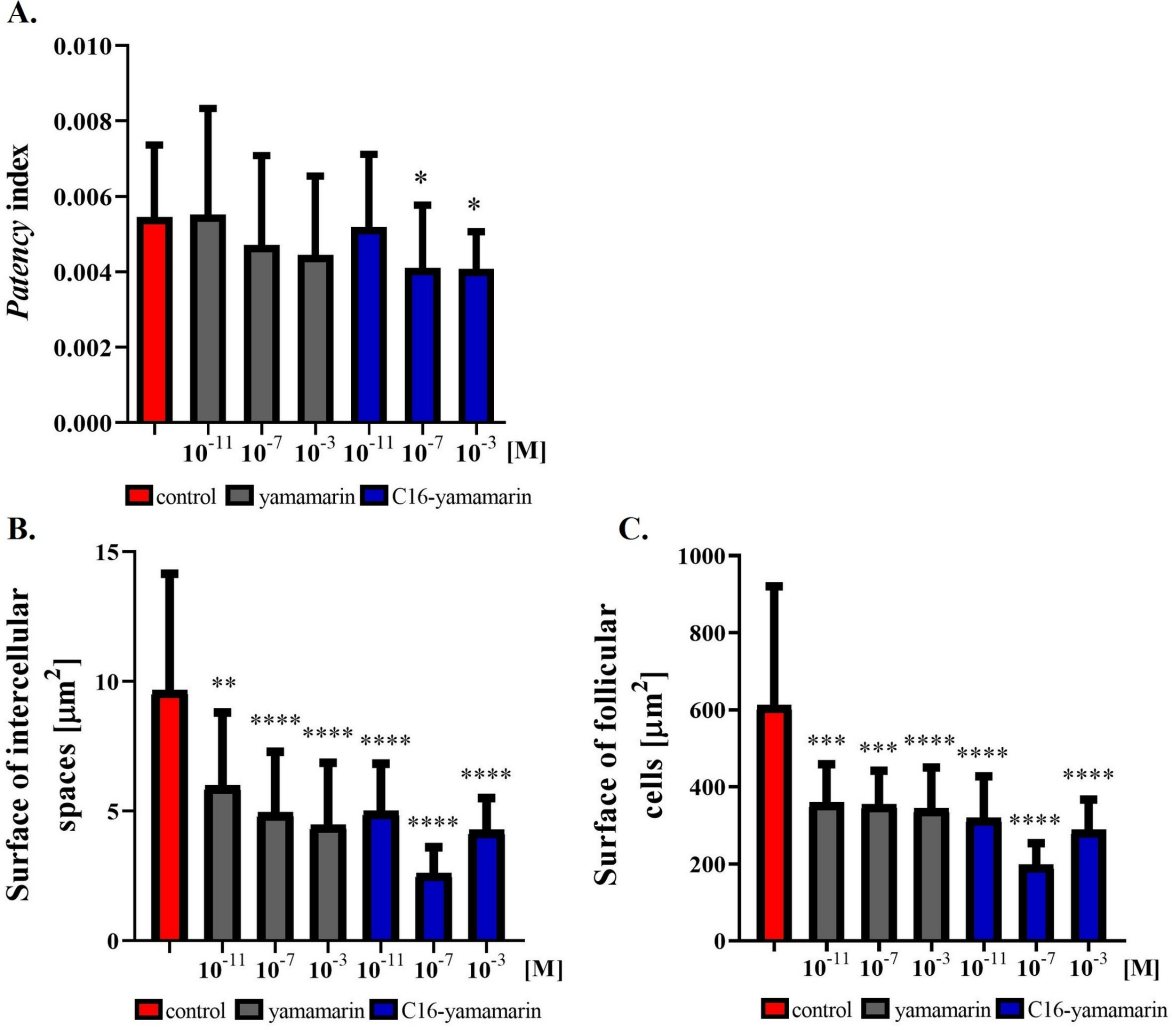
*Patency* index (A), surface of intercellular spaces (B) and surface of follicular cells. Statistically significant differences (one-way ANOVA with Dunn’s multiple comparisons test) in comparison to the control are indicated by asterisks *p* ≤ 0.1 (*), *p* ≤ 0.01 (**), *p* ≤ 0.001 (***), *p* <0,0001 (****), *n* ≥26.

Based on pictures from a confocal microscope (Fig 2), we calculated the value of the *patency* index, understood as the ratio of the surface of intercellular spaces to the sum of the area of the surrounding follicular cells, after the injection of yamamarin and C16-yamamarin (Fig 6). Statistically significant differences were observed after injection of C16-yamamarin at concentrations of 10^−7^ and 10^−3^ M. The other injections did not cause any significant changes in the index value. Interestingly, we observed changes in both the surfaces of intercellular spaces and follicular cells. Injection of yamamarin and C16-yamamarin in comparison to the control showed that the surface of follicular cells as well as intercellular spaces decreased. The most active was C16-yamamarin at concentration of 10^−7^ M after injection, of which the surface of intercellular space was reduced almost 3.7 times (average surface in control 9.65 μm^2^ and in C16-yamamarin 2.59 μm^2^) whereas the surface of follicular cells was reduced 3 times (average surface in control 611 μm^2^ and in C16-yamamarin 198 μm^2^) in comparison to the control.

### Influence of yamamarin on the expression of the gene encoding vitellogenin

**Fig 7:**
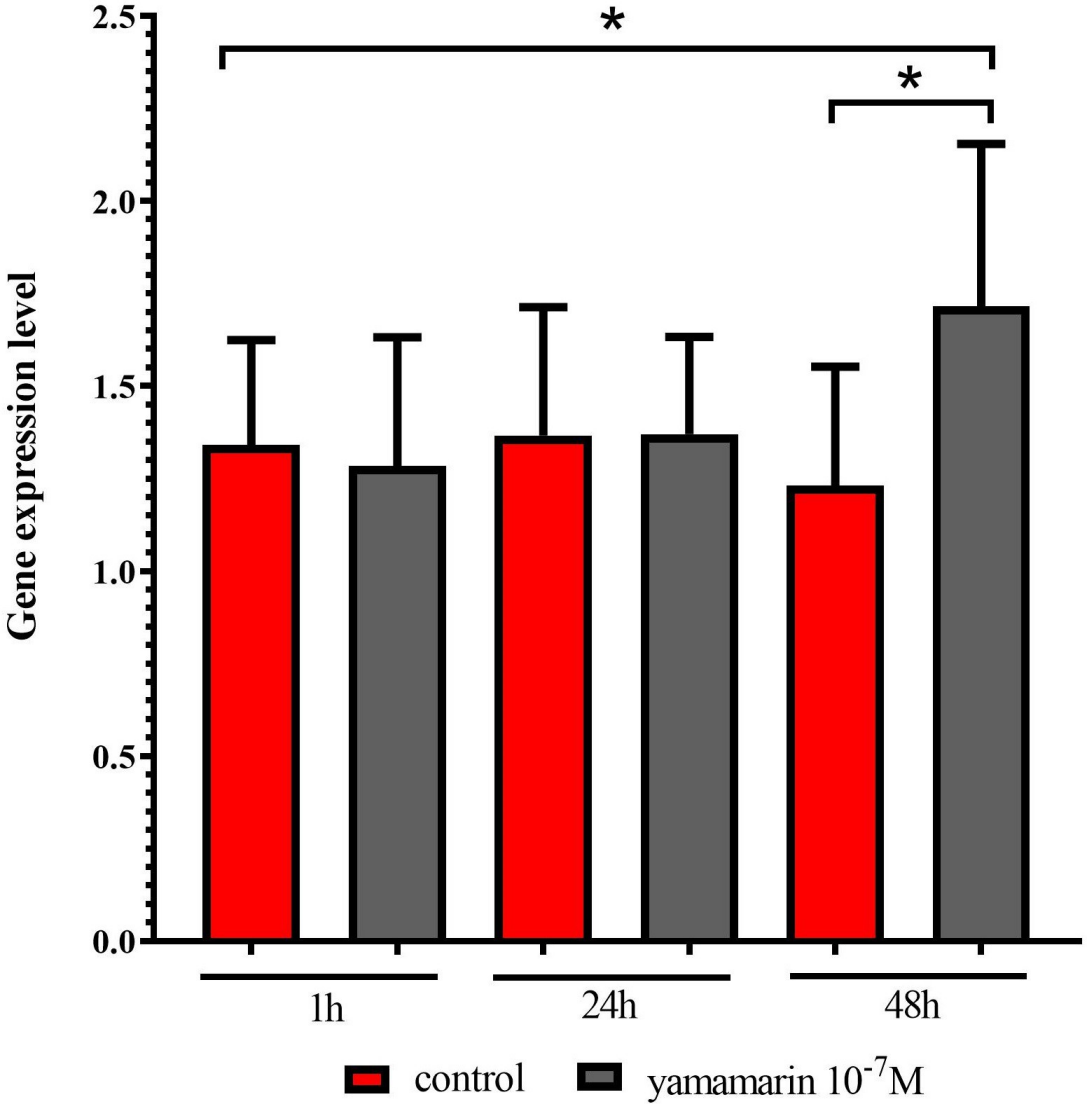
The expression level of *vitellogenin* in female fat body tissue 1 h, 24 h and 48 h after injection, was calculated by the ΔΔ*Ct* method (reference *syntaxin-1*). The data represent mean value ± SD from 4 independent biological (each biological sample consisted of tissue isolated from ≥ 10 females) and 3 technical repetitions. Statistical significance was tested with Kruskal-Wallis *H* = 15.49 and Mann-Whitney test (1 h control and 48 h yamamarin *U* = 37.0, 48 h control and 48 h yamamarin *U* = 25.0). Significant differences are indicated by asterisks as indicated: *p* ≤ 0.1 (*).

Vitellogenins are large (200–700 kDa) homologous phosphoglycolipoproteins that are synthesized in fat body tissue and become the major egg yolk protein [22]. Using qPCR, the effect of yamamarin at a concentration of 10^−7^ M on the number of mRNA copies in fat body tissue was analyzed and the expression level of the gene encoding vitellogenin was determined. A control injection of physiological saline was used. The gene encoding syntaxin-1 was used as a reference. When the results were compared with the 1 h after-injection control, statistically significant changes 48h after yamamarin injection were observed (*U* = 37.0). Statistically significant changes in the expression level of the *vitellogenin* gene were also observed between samples obtained 48 h after injection of physiological saline and yamamarin (*U =* 25.0) (Fig 7).

### The oviposition time and the number of eggs laid by females

**Fig 8:**
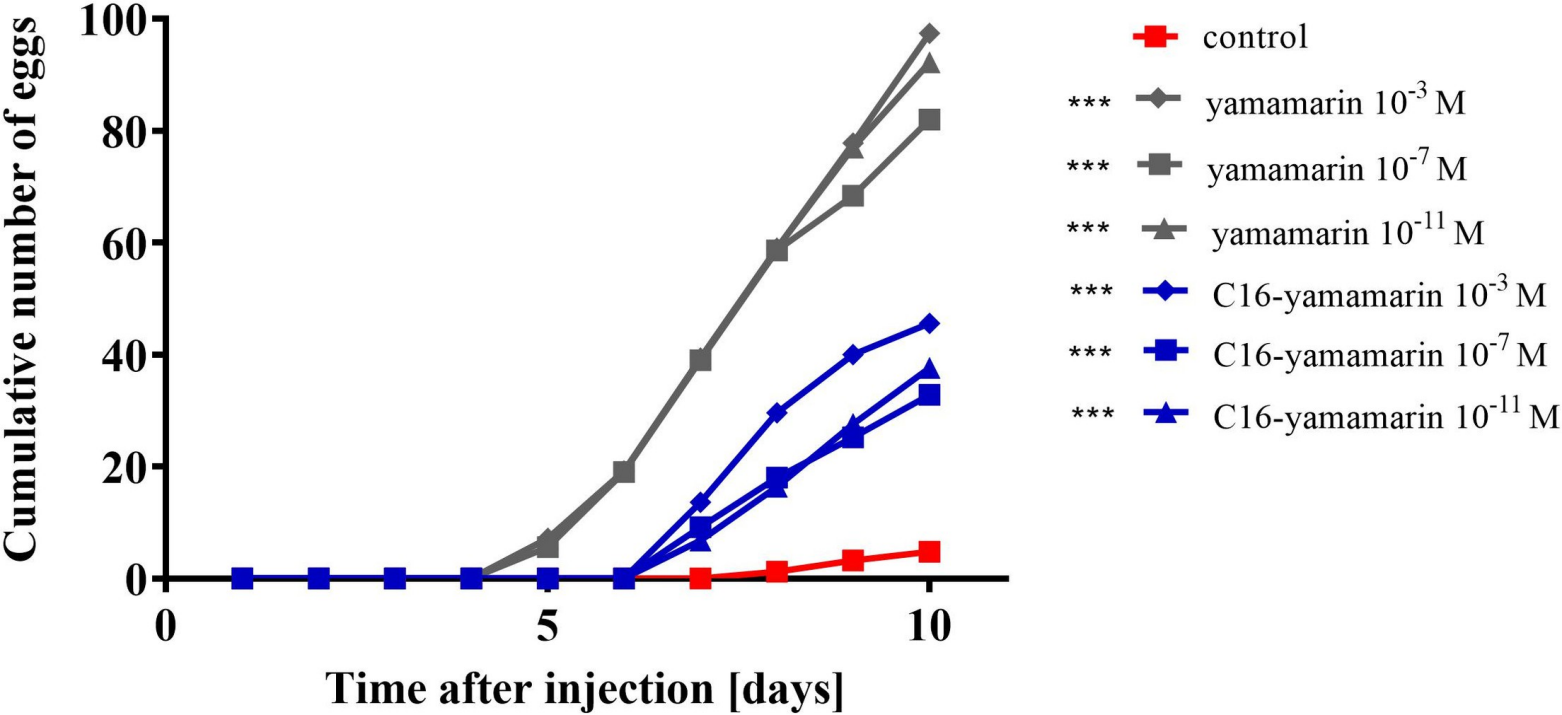
Oviposition starts time and the number of eggs laid by the females injected 1 day after eclosion with saline (control), yamamarin and C16-yamamarin. Statistically significant differences in comparison to the control (Spearman’s correlation coefficient) are indicated by asterisks *p* ≤ 0.001 (***).

Injection of 1-day old females with yamamarin and C16-yamamarin at each of the tested concentrations had an impact on the oviposition start time and the number of eggs laid by females (Fig 8). Control females started the oviposition process on day 8 during the first reproduction cycle. Compared to the control females, both yamamarin and its analog accelerated the oviposition start time and increased the number of eggs laid by females. Yamamarin induced a greater acceleration of the oviposition process than the analog. Females injected with yamamarin at all three concentrations laid their first eggs on the 5^th^ day of the reproductive cycle and those injected with the analog on the 7^th^ day.

Compared to the control, females injected with physiological saline, both yamamarin and C16-yamamarin showed a marked stimulating effect on egg production. The changes induced by both peptides in the number of eggs laid by females were statistically significant (for yamamarin *r* = 0,969; *p* ≤ 0.001, for C16-yamamarin *r* = 0,8876; *p* ≤ 0.001). There were, however, differences between the actions of the two peptides. More eggs were laid by females after yamamarin injection than after C16-yamamarin application. Yamamarin at each of the three injected concentrations elicited a similar response in egg production by females. Additionally, the largest change in egg production response was observed in females injected with 10^−3^ M concentrations of peptide and its analog.

### Duration of embryogenesis and hatchability

**Fig 9:**
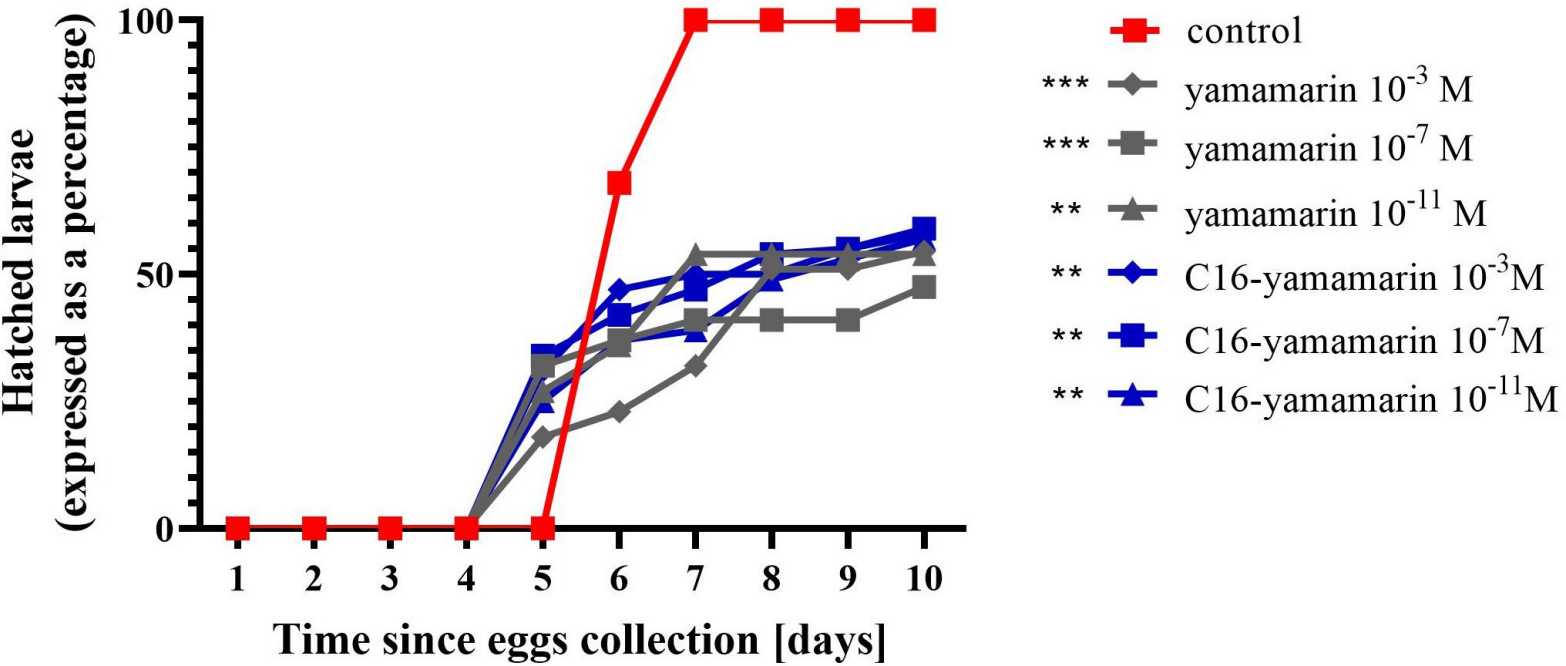
Duration of embryonic development and the hatchability of the next generation larvae from eggs laid by females injected with physiological saline (control), yamamarin and C16-yamamarin. Statistically significant differences (two-way ANOVA with Dunnett’s multiple comparisons test) in comparison to the control are indicated by asterisks *p* ≤ 0.001 (***), *p* ≤ 0.01 (**).

The effect of yamamarin and its analog on the duration of embryonic development and the hatchability of the next generation larvae were assessed on eggs collected at a time interval of 6 hours (fresh eggs) from 8-day-old females injected the day before with the test compounds. As a result of the action of these peptides, females produced eggs that were characterized by a shorter duration of embryogenesis and the decreased hatchability of the next generation larvae (Fig 9).

After injection of yamamarin and C16-yamamarin, the females laid eggs, from which, after 10 days of incubation, a significantly lower number of larvae hatched compared to the control (Fig 9). Approximately 50% of larvae hatched from eggs laid by females injected with yamamarin while approximately 60% hatched from eggs laid by females injected with the analog, and in both cases no dose-dependent response was observed for the concentrations of these peptides used. No morphological changes were observed in the newly hatched larvae.

### Duration of embryogenesis and hatchability of larvae from eggs treated topically with peptides

**Fig 10:**
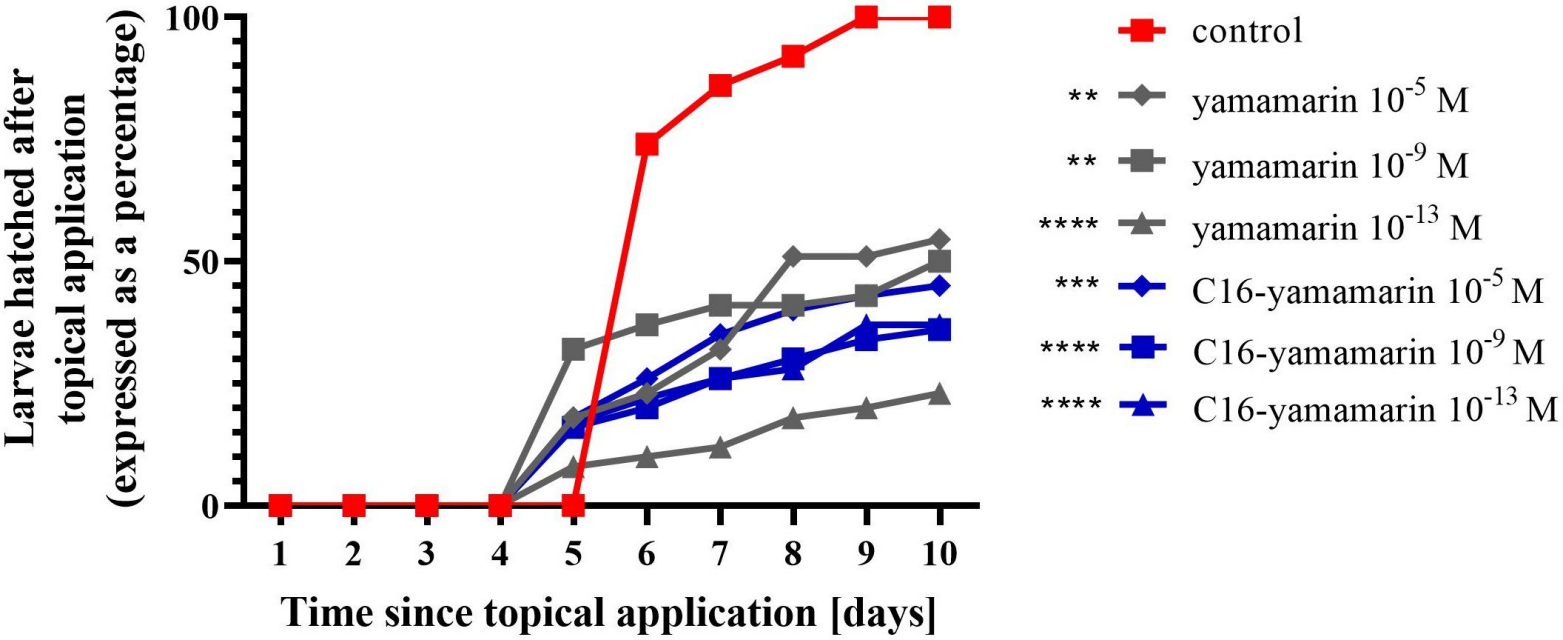
Duration of embryonic development and the hatchability of the next generation larvae from eggs, which were treated topically with physiological saline (control), yamamarin and C16-yamamarin. Statistically significant differences (two-way ANOVA with Dunnett’s multiple comparisons test) in comparison to the control are indicated by asterisks *p* ≤ 0.01 (**), *p* ≤ 0.001 (***), *p* <0,0001 (****).

In the conducted bioassay, the average time of embryonic development from fresh eggs in the control group was found to last 5 days and finally the larvae hatched from all the eggs (Fig 10). In this experiment, lower concentrations of peptides (10^−13^, 10^−9^ and 10^−5^ M) were used with reference to peptide concentrations in *in vivo* bioassays. Topical application of yamamarin and the C16-yamamarin analog to 1-day-old eggs resulted in a similar nature of the response during embryogenesis (Fig 10). Both tested peptides hasten larval hatchability for 1 day.

Topical applications of yamamarin and C16-yamamarin to fresh eggs at all three tested concentrations of these peptides resulted in a statistically significant decrease in the number of hatched larvae (Fig 10). Yamamarin at the two lowest concentrations (10^−13^ and 10^−9^ M) caused approximately 50% reduction in the number of hatched larvae, while at the highest concentration (10^−5^ M) this peptide induced a twice as strong effect. The analog at the lowest concentration (10^−13^ M) produced a similar reduction in the number of hatched larvae as the native peptide at low concentrations. The remaining two concentrations of the analog had a slightly stronger effect, but less than the effect of yamamarin at the highest concentration.

Additionally, we compared two methods of the administration of the tested compounds: by injection and by topical application. Analysis (two-way ANOVA with Tukey’s multiple comparisons test) did not show statistically significant differences between the methods of administration. Only the injection of yamamarin at a concentration of 10^−11^ M (*i*.*e*., the final concentration in the insect’s hemocel is to equal 10^−13^ M) and yamamarin at a concentration of 10^−13^ M given by topical application showed differences at the level of *p* < 0.0001. More active topical administration was more effective.

## Discussion

In this work, the effects of yamamarin peptide and its analog, C16-yamamarin, on the female reproductive system, its activity and embryonic development of *T*. *molitor* were investigated for the first time (Table 2). The results showed that the scope and specificity of the physiological action of yamamarin and its analog, constitute the starting point for the knowledge needed for further analysis of the structure-activity relationship and the synthesis of strong agonists and antagonists that can be used as bioinsecticides or biopharmaceuticals.

**Table 2:** Summary of the effects caused by yamamarin and palmitic acid-conjugated yamamarin.

Concentration [M]	injection												topical application		injection	
	follicular epithelium formation		*patency* index		average volume of terminal oocyte		oviduct enlargement		the number of eggs laid by females		larvae hatchability		larvae hatchability		expression of gene encoding vitellogenin	
	Y	C16-Y	Y	C16-Y	Y	C16-Y	Y	C16-Y	Y	C16-Y	Y	C16-Y	Y	C16-Y	time	Y (10^−7^ M)
10^−11^	**NC**	**NC**	**NC**	**NC**	**I**	**NC**	**NC**	**NC**	**I**	**I**	**D**	**D**	**D**	**D**	1 hour	**NC**
10^−7^	**NC**	**NC**	**NC**	**D**	**I**	**NC**	**NC**	**NC**	**I**	**I**	**D**	**D**	**D**	**D**	24 hours	**NC**
10^−3^	**NC**	**NC**	**NC**	**D**	**NC**	**NC**	**I**	**NC**	**I**	**I**	**D**	**D**	**D**	**D**	48 hours	**I**
LEGEND	**Y** yamamarin		**C16-Y** C16-yamamarin		**NC** no changes		**I** increase		**D** decrease							

In our studies, we showed that yamamarin stimulated the development of the ovaries, accelerating the growth and maturation of terminal oocytes, which may be due to the influence of the injected peptides on ecdysteroid levels, including the hormone ecdysone, which through the receptor ecdysteroid (EcR) directly influences the development of the ovary, vitellogenesis and the growth and maturation of oocytes [23, 24]. Studies carried out on the ovaries of *D*. *melanogaster* showed that ecdysone causes changes in the expression level of genes that regulate the growth and degeneration of emerging egg cells [25, 26]. In the studies conducted, we observed ovariole terminal vesicles with well-developed follicular epithelium and intercellular spaces. Both the average surface of intercellular spaces and follicular cells were significantly smaller than the average surface of intercellular spaces and follicular cells in the control group; however, this difference did not translate into a decrease in the *patency* index. Injections of yamamarin increased the volume of terminal oocytes. Interestingly, C16-yamamarin did not cause changes in that parameter. Research conducted by Parthasarathy, Sun [27], Wu, Yang [28] and Mordue [29] showed that the vitellogenesis process is regulated by the juvenile hormone (JH) produced in *corpora allata*, so perhaps an increase in the volume of terminal oocytes by yamamarin is the result of its interaction with brain endocrine cells synthesizing the neurohormone allatotropin, which stimulates *corpora allata* to increase juvenile hormone synthesis and/or release [30, 31]. Another reason for the increased volume of terminal oocytes may be the metabolic activity of juvenile hormone (JH), which stimulates protein synthesis in fat body cells, which is a reservoir of energy in the form of glycogen, triglycerides, and proteins [32, 33]. Metabolism, synthesis and utilization of lipids and proteins are extremely important both for growth and reproduction [32]. Because fat body tissue is a place of integration of many signals coming from all over the insect body, many of them are subject to hormonal control, such as ecdysone, which interacts with juvenile hormone. Their mutual balance is essential to carry out the processes necessary during development and metamorphosis, so it is also possible that the tested peptides influence not strictly on the activity of juvenile hormone but also on the activity of ecdysteroids or affect their mutual dependence however, to confirm these speculations, further and more detailed research is necessary [34]. Moreover, an increased volume of terminal oocytes may result from an increased expression of the gene encoding vitellogenin (yolk precursor) in the fat body and a greater supply of this protein during the period of vitellogenesis to developing oocytes [35], which is important because the lipid and protein content in oocytes is in 99% effect of fat body tissue activity, as the ability to synthesize fatty acids *de novo* in ovaries is strongly reduced (1%) [32]. The effect of an increased supply of lipids and proteins into oocytes might also be an effect of yamamarin on the transportation system, such as lipophorins, which may cause this system to be more effective [36, 37]. The observed effect of the increased volume of terminal oocytes might be the result of the action of yamamarin and its conjugate on the activity of JH, which influences the increased synthesis of vitellogenin proteins, that are then released into the hemolymph to make up the yolk and increase the sequestration into the oocyte, which is considered an extremely dynamic process [38, 39]. As it is impossible to work alone, fat body tissue and its products are part of many metabolic pathways, such as the rapamycin pathway [38–40], as was shown in mosquito *Aedes aegypti*. In mosquitos, after a blood meal, the synthesis of *Vg* was upregulated, which was an effect of reactions that began at the fat body plasma membrane, especially amino acid transporters, leading to activation of the rapamycin pathway and resulting in increased translation of *Vg* [40]. The data obtained showed an increased expression level of vitellogenin 48 h after yamamarin application. An increase in total RNA in *T*. *molitor* ovary tissue during the first gonadotropic cycle was also observed by Ullmann [11]. Stating that this process was time dependent, she observed the smallest amount of RNA in oocytes after approximately 24 hours from the beginning of the vitellogenesis phase [11], which is supported by our study showing that the maximum level of vitellogenin gene expression in fat body tissue was observed 2 days after the beginning of vitellogenesis. In *A*. *aegypti* mosquitos, a peak of vitellogenin was observed 30 h after blood meal reception. In our research, to obtain the effects of *Vg* expression, we used females injected on the first day after eclosion, and we need to remember that during the pupal stage and pupation insects do not eat, which is why we might suspect that yamamarin and its conjugate affect the activity of transporters in fat body tissue, which causes changes in ramapycin (mTOR) activity [32, 40]. Our experiments showed that the injection of the peptides increased the number of eggs laid by female *T*. *molitor* and that both peptides accelerated the egg-laying process. An increased number of laid eggs may be a result of a stimulating effect on the course of the oogenesis process, such as intensive cell division in the germarium and maturation of oocytes in the previtellogenesis and vitellogenesis phase, as well as accelerating ovulation of eggs and stimulating their faster passage through the lateral oviduct and the common oviduct tube. Yamamarin and its C16 analog may possess activity similar to the activity of allatostatins, such as Grybi-MIP1, Dippu-AST1 or Trica-ASTC, which in heterologous systems showed a stimulating effect on oviducts of *T*. *molitor* and *Zophobas atratus* beetles [41, 42], which translated into faster passage of oocytes. Further experiments showed that the influence of the tested compounds on the processes related to the reproduction of *T*. *molitor* is more comprehensive and concerns not only the number of eggs laid but also the hatchability of the larvae, which may largely characterize changes in the egg fertilization process when the injection variant is analyzed. Increased numbers and speeding up the time of laying eggs and at the same time reduced hatchability of larvae suggest that the tested peptides caused the laying of large numbers of unfertilized eggs or that the eggs did not accumulate substances necessary for the development of the embryo, such as vitellogenin (which suggests no changes in *patency* with a simultaneous increase in the volume of terminal oocytes), in return, *e*.*g*. triglycerides or water were accumulated in the eggs, which caused an increase in the volume of terminal oocytes, at the same time not being sufficient for the development of the insect. The effect of laying unfertilized eggs may in be turn eliciting the functioning of endocrine glands—*corpora allata*, and stimulating them to increase the release of JH, which was found *e*.*g*., in the female bug *Podisus nigrispinus* [43]. Research conducted by Sugime, Watanabe [44] on *D*. *melanogaster* indicated that an increased supply of JH with food resulted in the submission of female flies and an increased number of unfertilized eggs. Another reason for the laying of unfertilized eggs may be the result of a disturbance in sperm release from the seminal reservoir, a nonsynchronous release of sperm and oocytes or changes in eggshell structures, the presence of which is needed for fertilization, the micropyle [45]. There is also the possibility that the tested peptides cause changes in the egg structures needed for respiration (outer structure called the aeropyle or inner structures that provide transport for oxygen inside the egg). Modifications of the chorion structure, which surrounds the eggshell, may disturb efficient gas exchange, causing gas poisoning of the embryo, or might cause increased water loss during respiration, which will lead to above-average drying of the embryo [46]. Jacobs, Rezende [47] conducted research that concerns another structure surrounding the embryo–the serosa, which in fact is the extraembryonic membrane that tucks in the embryo and its function is secreting the cuticle. In those studies, they injected the beetle *Tribolium castaneum*, which is closely related to *T*. *molitor*, with *zerknüll1* RNAi, which causes the serosa structure not to form, postpones the to lower humidity of the embryo and results in a decreased hatching ratio [47]. When we compare changes caused by those modifications, we might suppose that yamamarin and its analog, which, thanks to the addition of saturated fatty acids, possess an increased ability to penetrate biological membranes. In the analysis of mechanisms in reproductive disorders in *T*. *molitor* caused by the tested peptides, it was necessary to investigate whether these compounds had been not only ootoxic but also embryotoxic. The experiments performed with topical application of yamamarin and C16-analog on eggs, which were conducted to check the direct effect of peptides on embryogenesis, resulted in a similar reduction in hatchability as well a shortened period of embryonic development and accelerated larval hatching. The results obtained thus indicate that the tested compounds are embryotoxic to the developing embryo after penetrating through the chorion sheath or after passing through into the egg through the micropyle. Comparison of ways of administration did not show statistically significant differences. One exception was the injection of yamamarin at a concentration of 10^−11^ M (*i*.*e*., the final concentration in the hemocel of the insect is equal to 10^−13^ M) and yamamarin at a concentration of 10^−13^ M given by topical application, when the topical method turned out to be more effective. The mechanism of their penetration and the degree of toxic action is determined by the size of the molecule and the hydrophobic properties of the *N*-terminus of tested peptides and their mimetics [48, 49]. In this case, C16-yamamarin was more active, which might be because it is a 5 amino acid molecule conjugated with palmitic acid. These changes resulted in increased potential of hydrophobic properties at the *N*-terminus of the amino acid chain, and it might penetrate more easily into eggs, disrupting the development of the embryo. We wondered, why the observed effects showed low dose-dependence, and in some cases, the lower concentration evoked a stronger response. The most possible explanation of this phenomenon may be that the studies were carried out mainly *in vivo*, so we do not know if the peptide after application induces a given effect directly by affecting only the tested tissues, or indirectly influencing other tissues/systems (for example, the neuroendocrine system, which is involved in the neuroendocrine regulation of metabolism and reproductive processes), which secondarily affects tested tissues. This case is most likely for compounds that cause pleiotropic effects. Of course, the tested compounds seem to be more likely to act simultaneously in both ways, and we observed cross-talk between direct and indirect effects. Moreover, different tissues/organs can have different sensitivities to the tested compounds and the final, general effect results from other effects “on the lower levels”. Taking all these findings into consideration, we might assume that the effect of yamamarin and its acid analog might be correlated with embryotoxicity of peptides, not with disruption of fertilization. The gonadotropic effects of yamamarin and its peptidomimetic might be multidirectional and marked by their diversified influence on the process of vitellogenesis, oogenesis and ovulation. In addition to influencing the processes, physiological reproduction-related peptides have an even broader spectrum of biological activity, affecting embryogenesis processes. The embryotoxic nature of the action of these compounds after their application indicates the potential possibilities of their use as bioinsecticides or as a compound of natural origin (biopharmaceutical) causing a reduction in the rate or inhibition of intense cell division taking place, among others in cancer cells [50]. The peptide appears to be a candidate for the further scientific description and development of compounds that might be used or might serve as a model for peptide and their analog synthesis to limit the number of pests. Certain fact is that results obtained from presented experiments clearly showed direction that should be taken when chemically modified peptide molecules (peptidomimetics) are designed. Peptidomimetics may be an alternative method of pest control and could eventually replace traditional synthetic insecticides with low species-specificity and highly harmful effects on nontarget organisms [51–53]. To date, in the design and synthesis of identified peptidomimetics, modifications such as attaching fatty acid molecules to native peptides have increased their hydrophobic properties, ability to penetrate the cuticle, half-life of the active form or resistance to proteases [51, 52, 54, 55]. Therefore, the obtained results will fill the gap in knowledge and might contribute to creating the basis to develop effective peptidomimetics in the future.

## Supporting information

S1 File Primer amplification efficiency calculations. (XLSX)
